# Rac1 Pharmacological Inhibition Rescues Human Endothelial Dysfunction

**DOI:** 10.1161/JAHA.116.004746

**Published:** 2017-02-28

**Authors:** Albino Carrizzo, Carmine Vecchione, Antonio Damato, Flavio di Nonno, Mariateresa Ambrosio, Franco Pompeo, Enrico Cappello, Luca Capocci, Mariangela Peruzzi, Valentina Valenti, Giuseppe Biondi‐Zoccai, Antonino G. M. Marullo, Silvia Palmerio, Roberto Carnevale, Chiara C. Spinelli, Annibale A. Puca, Speranza Rubattu, Massimo Volpe, Junichi Sadoshima, Giacomo Frati, Sebastiano Sciarretta

**Affiliations:** ^1^ IRCCS Neuromed Pozzilli (IS) Italy; ^2^ Department of Medicine and Surgery University of Salerno Baronissi (SA) Italy; ^3^ Department of Medical‐Surgical Sciences and Biotechnologies Sapienza University of Rome Polo Pontino Latina Italy; ^4^ Department of Imaging Bambino Gesù Children Hospital IRCCS Rome Italy; ^5^ IRCCS Multimedica S.p.A Milan Italy; ^6^ Department of Clinical and Molecular Medicine Sapienza University of Rome Italy; ^7^ Department of Cell Biology and Molecular Medicine Rutgers New Jersey Medical School Newark NJ

**Keywords:** cardiovascular disease, endothelial dysfunction, nitric oxide, oxidative stress, Endothelium/Vascular Type/Nitric Oxide, Oxidant Stress

## Abstract

**Background:**

Endothelial dysfunction contributes significantly to the development of vascular diseases. However, a therapy able to reduce this derangement still needs to be identified. We evaluated the effects of pharmacological inhibition of Rac1, a small GTPase protein promoting oxidative stress, in human endothelial dysfunction.

**Methods and Results:**

We performed vascular reactivity studies to test the effects of NSC23766, a Rac1 inhibitor, on endothelium‐dependent vasorelaxation of saphenous vein segments collected from 85 subjects who had undergone surgery for venous insufficiency and from 11 patients who had undergone peripheral vascular surgery. The endothelium‐dependent vasorelaxation of the varicose segments of saphenous veins collected from patients with venous insufficiency was markedly impaired and was also significantly lower than that observed in control nonvaricose vein tracts from the same veins. Rac1 activity, reactive oxygen species levels, and reduced nicotine adenine dinucleotide phosphate (NADPH) oxidase activity were significantly increased in varicose veins, and NSC23766 was able to significantly improve endothelium‐dependent vasorelaxation of dysfunctional saphenous vein portions in a nitric oxide–dependent manner. These effects were paralleled by a significant reduction of NADPH oxidase activity and activation of endothelial nitric oxide synthase. Finally, we further corroborated this data by demonstrating that Rac1 inhibition significantly improves venous endothelial function and reduces NADPH oxidase activity in saphenous vein grafts harvested from patients with vascular diseases undergoing peripheral bypass surgery.

**Conclusions:**

Rac1 pharmacological inhibition rescues endothelial function and reduces oxidative stress in dysfunctional veins. Rac1 inhibition may represent a potential therapeutic intervention to reduce human endothelial dysfunction and subsequently vascular diseases in various clinical settings.

## Introduction

Endothelial dysfunction leads to the development of arterial vessel inflammation, remodeling, and atherosclerosis and, by this means, is significantly associated with an increased incidence of ischemic heart disease, peripheral artery disease, and stroke.^1^, ^2^ However, endothelial dysfunction also appears to contribute significantly to the development of vein‐related diseases.^3^, ^4^, ^5^, ^6^, ^7^, ^8^ Venous endothelial function is significantly affected by cardiovascular risk factors such as diabetes mellitus and a smoking habit, which are known to impair arterial function and to promote cardiovascular diseases.^4^, ^9^, ^10^, ^11^ Endothelial dysfunction appears to be involved in the development of deep vein thrombosis^3^ and vein insufficiency.^5^ In addition, an impairment of endothelial function could be implicated in the development of venous graft remodeling, atherosclerosis, and failure in subjects undergoing myocardial surgical revascularization through bypass implantation.^6^, ^7^, ^8^ This evidence indicates that a pharmacological intervention able to restore venous endothelial function might represent a potential therapeutic strategy to reduce vein‐related diseases. Unfortunately, a suitable pharmacological target to reduce endothelial dysfunction in human subjects still needs to be identified.

Oxidative stress is considered to be the main cause of endothelial dysfunction, mostly through the reduction of nitric oxide bioavailability,^1^, ^2^ which is one of the most important mediators of the physiological properties of endothelial cells. Among the sources of reactive oxygen species (ROS), the NADPH oxidase (NOX) family plays a major role in the genesis of endothelial oxidative stress.^12^, ^13^, ^14^, ^15^, ^16^ However, the role of NOXin the development of venous endothelial dysfunction remains to be fully clarified. In addition, the most suitable way to inhibit pathological activation of NOX in the endothelium during stress, particularly in human subjects, still needs further elucidation.

The small GTP‐binding protein Rac1 might represent a suitable target to inhibit maladaptive NOX activity.^17^, ^18^ Rac1 was previously found to activate NOX in endothelial cells and to promote ROS production in response to stress.^17^, ^18^ Rac1 increases vascular permeability and inflammation.^17^, ^18^ In addition, we previously found that Rac1 inhibition rescues both diabetes mellitus‐induced and high blood pressure–induced arterial endothelial dysfunction.^19^, ^20^ However, the role of Rac1 in the development of human endothelial dysfunction, particularly in veins, is unknown.

Thus, the aim of this study was to evaluate for the first time the effects of pharmacological Rac1 inhibition in human endothelial dysfunction. For this purpose, we conducted ex vivo studies with dysfunctional saphenous veins collected from patients with chronic vein insufficiency and saphenous veins harvested from patients who had undergone bypass surgery for peripheral chronic ischemia.

## Methods

### Human Studies

#### Study Design

We conducted our experiments on intact saphenous vein portions removed from 2 groups of subjects: (1) 85 subjects who underwent surgery for chronic venous insufficiency and varicose veins (group 1) and (2) 11 subjects who underwent bypass surgery for peripheral chronic ischemia (group 2). Inclusion criteria were (1) severe saphenous vein insufficiency and/or varicose veins (group 1),or chronic peripheral ischemia suitable for surgical revascularization by means of saphenous vein graft (bypass) (group 2); (2) age ≥18 and ≤85 years; (3) elective surgery. Exclusion criteria were (1) acute and chronic inflammatory diseases; (2) immunological disorders; (3) active infections; (4) previous organ transplantation; (5) any pharmacological therapy able to modulate endothelial function and/or either Rac1 or NOX activity within the previous month; or (6) any surgical complication.

Vessels were collected immediately after surgical harvesting and transported to the laboratory in ice‐cold Krebs‐HEPES buffer to perform vascular reactivity studies.

### Vein Preparation and Ex‐Vivo Experiments

The study protocol was approved by all local ethics committees of IRCCS Neuromed and done in accordance with the Declaration of Helsinki. All participants gave written informed consent. Institutional review board approval was obtained from IRCCS Neuromed (No. 20160106‐1006).

Hypercholesterolemia was considered as total plasma cholesterol, <240 mg/dL smoking was considered as current or within the last 6 months, and hypertension as current treatment with antihypertensive agents.^21^, ^22^, ^23^ The vessels were supplied before the dilation procedure and vasodilator application. Care was taken during harvesting of the vessels so as not to stretch or touch the endothelial surface. The vessel species were placed immediately into cold (4°C) Krebs Ringer solution of the following composition (mmol/L): NaCl 118, KCl 4.7, KH_2_PO_4_ 1.2, NaHCO_3_ 25, MgSO_4_·d7H_2_O 1.2, CaCl_2_ 2.5, glucose 11.1, and disodium EDTA 0.026. The vessels were cleaned of adherent connective tissues and cut into rings 3 to 4 mm in length. Rings were suspended between 2 stainless steel L‐shaped hooks in a 10‐mL jacketed organ bath containing Krebs Ringer solution at 37°C and aerated with 95% O_2_ and 5% CO_2_. One hook was fixed to a micrometric manipulator allowing adjustments in resting tension of the rings, and the other was connected to a force displacement transducer (WPI Instruments, Sarasota, FL) for the measurement of isometric force. The optimal point of the length‐tension relation had been previously described.^24^

At the end of an equilibration period of 2 hours, the viability of the vessel segments was checked through the evaluation of the cumulative dose‐response curves to norepinephrine (10^−9^ to 10^−6^ mol/L) and contraction to 40 mmol/L KCl, which had to be reproducible at least 2 times. To study the endothelium‐dependent vasorelaxation, saphenous veins were precontracted with 10^−9^ to 10^−6^ mol/L of phenylephrine to obtain a contraction that corresponds to 70% to 90% of maximal contractions observed with KCl. When the constriction reached a stable plateau, increasing doses of acethylcoline (10^−9^ to 10^−6^ mol/L) were added.

Cumulative dose‐response curves to acetylcholine in saphenous veins were repeated in the presence of NSC23766 (30 μmol/L), L^G^‐nitro‐l‐arginine (L‐NAME, 300 μmol/L) or polyethylene glycol superoxide dismutase (PEG‐SOD, 500 U/mL), which were added to the bathing medium 30 minutes before acetylcholine administration. Responses to acetylcholine were assessed in each vessel before and after administration of NSC23766. In time‐matched control experiments, we determined whether the precontraction induced by phenylephrine is stable enough for the period required to construct the cumulative relaxation curves to acetylcholine.

### Reagents and Biochemical Analyses

All drugs used were purchased from Sigma (St. Louis, MO) or Tocris Bioscience (Bristol, UK). A stock solution of phenylephrine or acetylcholine was freshly prepared to prevent oxidation. NSC23766 and L‐NAME were dissolved in distilled water.

### Immunoblotting

Vessels were solubilized in lysis buffer containing 20 mmol/L Tris‐HCl, 150 mmol/L NaCl, 20 mmol/L NaF, 2 mmol/L sodium orthovanadate, 1% Nonidet, 100 μg/mL leupeptin, 100 μg/mL aprotinin, and 1 mmol/L phenylmethylsulfonyl fluoride. Then, samples were left on ice for 30 minutes and centrifuged at 10 621*g* for 20 minutes, and the supernatants were used to perform immunoblot analysis. Total protein levels were determined using the Bradford method. Rac1 activity was determined using a commercially available kit (Cell BioLabs Inc, San Diego, CA, STA‐401‐1) as described below. Forty micrograms of proteins were resolved on 10% SDS‐PAGE, transferred to a nitrocellulose membrane, and immunoblotted with anti‐Rac1‐GTP (1:1000, Cell BioLabs) or anti‐Rac1 (1:1000, Abcam, Cambridge, UK); with anti–RhoA‐associated kinase 1 (ROCK1) (1:1000 (abcam); with anti‐p–endothelial nitric oxide synthase (eNOS) phosphorylated on serine 1177 (1:800, Abcam) or anti‐total‐eNOS (1:800, Abcam); or with anti‐β‐actin (1:1000, Cell Signaling, Danvers, MA). Horseradish‐peroxidase–conjugated secondary antibodies were used at 1:3000 dilution (Bio‐Rad Laboratories, Hercules, CA). Protein bands were detected by ECL Prime (Amersham Biosciences, Little Chalfont, UK), and densitometry analysis was performed using Quantity One software (Bio‐Rad Laboratories).

#### Detection of Endothelial Nitric Oxide Synthase Dimer and Monomer

Low‐temperature SDS‐PAGE (LT‐PAGE) was performed for detection of SDS‐resistant endothelial nitric oxide synthase (eNOS) dimer and monomer, as described previously.^25^

### Rac1*‐*GTP Pull‐Down Experiments

Tissues were lysed in a buffer containing NP‐40 equipped by kit STA‐401‐1 (Cell Biolabs Inc, San Diego, CA). The p21‐binding domain of p21‐activated protein kinase bound to agarose beads was added, and active Rac1, binding PAK1, was separated by repetitive centrifugation and washing. Then, the specimens were boiled in Laemmli buffer and subjected to SDS‐PAGE, and Rac1 was quantified by Western blot analysis. In detail, Rac1‐GTP was detected with the monoclonal antibodies anti‐Rac1‐GTPγ (1:800; STA‐401‐1, Cell Biolabs Inc) and total Rac1 with monoclonal anti‐Rac1 (1:1000; Abcam). Densitometry analysis was performed using Quantity One software (Bio‐Rad Laboratories). The amount of Rac1‐GTP was normalized to the total amount of Rac1 in tissue lysates for the comparison of Rac1 activity (GTP‐bound Rac1) among different samples.

### Evaluation of ROS Production

#### Dihydroethidium Staining

Dihydroethidium (DHE) was used to evaluate the levels of oxidative stress in saphenous veins as previously described.^26^ Briefly, vessels were stained with 10 μmol/L DHE for 20 minutes and observed under a fluorescence microscope (Zeiss, Oberkochen, Germany). Images were acquired by a digital camera system.

#### NADPH Oxidase Activity Measurement

NOX activity in saphenous veins was measured before and after NSC23766 treatment. Vein segments (3 cm) were placed in a chilled modified Krebs/HEPES buffer containing (mmol/L) NaCl 99.01, KCl 4.69, CaCl_2_ 1.87, MgSO_4_ 1.20, K_2_HPO_4_ 1.03, NaHCO_3_ 25.0, sodium HEPES 20.0, and glucose 11.1, pH 7.4. Periadventitial tissue was carefully removed, and the vessels were repeatedly washed to remove adherent blood cells. A 10% vessel homogenate was prepared in a 50 mmol/L phosphate buffer containing 0.01 mmol/L EDTA. The homogenate was then subjected to low‐speed centrifugation (1000*g*) for 10 minutes to remove unbroken cells and debris. Supernatants (20 μL) were added to glass scintillation vials containing 5 μmol/L lucigenin in 2 mL phosphate buffer. The chemiluminescence that occurred over the ensuing 5 minutes in response to addition of 100 μmol/L NADPH was recorded (Beckman LS6500 Multipurpose Scintillation counter; Beckman Coulter, Fullerton, CA). In preliminary experiments, homogenates alone without addition of NADPH gave only minimal signals. Furthermore, NADPH did not evoke lucigenin chemiluminescence in the absence of homogenate. In some experiments, 30 U/mL of heparin‐binding SOD was added to determine the SOD‐inhibitable activity. This is a recombinant form of SOD that contains a heparin‐binding domain that permits a close association of SOD with cell membranes, obviating electrostatic repulsion of SOD. The subcellular fraction of cytosol and membranes were obtained as previously described.^27^

### Statistical Analysis

All data are presented as mean±SEM. For continuous variables, we used a t test to compare 2 independent groups. When 2 samples from the same individual were compared, a paired t test was used. When more than 2 independent groups were compared, we used a 1‐way ANOVA analysis followed by Bonferroni post hoc test. Finally, in order to analyze the effects of our treatments on endothelium‐dependent vasorelaxation in response to increasing doses of acetylcholine, we performed a 2‐way repeated‐measures ANOVA with Bonferroni post hoc test for multiple comparisons.

A *P* value of less than 0.05 was considered statistically significant. All statistical analyses were conducted with Prism statistical software (Graphpad, La Jolla, CA).

## Results

**Figure 1:**
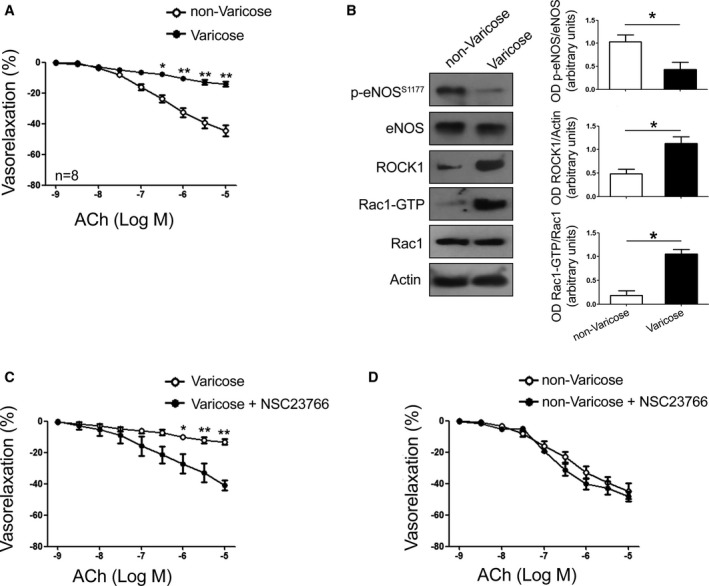
Rac1 inhibition rescues endothelial function in dysfunctional human saphenous veins. A, Dose‐response curves of relaxation of human saphenous vein healthy tracts (non‐Varicose) and varicose saphenous vein portions (Varicose) collected from the same patients in response to increasing doses of acetylcholine (ACh). The response obtained was expressed as the percentage of the isometric tension produced by 80 mmol/L KCl. Data are given as mean±SEM (n=8). *P<0.05; **P<0.01. B, Representative immunoblot analysis from nonvaricose and varicose portions of saphenous veins collected from the same patients. Data are given as mean±SEM *P<0.05 (n=4). C and D, Dose‐response relaxation curves of human varicose and nonvaricose saphenous veins, with and without Rac1 inhibitor NSC23766 30 μmol/L, in response to increasing doses of acetylcholine (ACh). Data are given as mean±SEM (n=8 for each group). OD indicates optical desnsity; p‐eNOS, p‐endothelial nitric oxide synthase; ROCK1, RhoAassociated kinase 1.

In order to study the role of Rac1 in the development of human endothelial dysfunction, we conducted ex vivo experiments on saphenous vein portions obtained from 85 subjects who underwent saphenectomy due to chronic venous insufficiency and/or varicose veins. The clinical characteristics of the study subjects are reported in Table. In the saphenous veins collected from 12 of these subjects it was still possible to isolate healthy tracts with preserved structure (ie, not dilated, not varicose, and not thickened) and separate them from the varicose portions (Table). We found that the endothelium‐dependent vasorelaxation of varicose vein segments was significantly lower than the vasorelaxation of the healthy venous segments isolated from the same veins (Figure 1A). This evidence indicates that varicose saphenous veins are dysfunctional, further supporting their use as a model of venous endothelial dysfunction. Mechanistically, Rac1‐GTP content, a marker of Rac1 activation, was found to be significantly higher in the varicose vein portions as compared to the nonvaricose ones. On the other hand, eNOS phosphorylation on serine 1177, a marker of activation of the enzyme, was significantly reduced in the varicose tracts. Accordingly, the expression level of ROCK1, an eNOS inhibitor, was significantly increased in the altered vein portions as compared to the healthy ones (Figure 1B). Overall, these data indicate that Rac1 is activated in dysfunctional vein tracts, and this is associated with reduced eNOS activity and increased ROCK1 expression level.

**Table 1:** Clinical and Demographic Characteristics of Patients Undergoing Surgery for Chronic Venous Insufficiency and of Patients in Whom It Was Possible to Separate Healthy Saphenous Vein Tracts From Varicose Portions and of Patients Undergoing Peripheral Bypass Surgery

	Patients With Chronic Venous Insufficiency	Healthy Saphenous Vein Tracts From Varicose Portions	Peripheral Bypass Surgery
Population, n	85	12	11
Age average, y	57±7	58±2	58±6
Risk factors, n (%)			
Pathology			
Hypertension	38/85 (44)	5/12 (41.6)	5/11 (45.5)
Dyslipidemia	23/85 (27.7)	4/12 (33.3)	4/11 (36.4)
Cardiopathy	10/85 (11.7)	4/12 (41.6)	5/11 (45.5)
Diabetes mellitus	9/85 (10.5)	3/12 (25)	3/11 (27.3)
Smoker	26/85 (33.3)	5/12 (41.6)	4/11 (36.4)
COPD	23/85 (27)	4/12 (33.3)	5/11 (45.5)
Hepatopathy	5/85 (5)	0/12	0/11 (0)
Dysthyroidism	13/85 (15)	2/12 (16.6)	2/11 (18.2)
Previous surgery	54/85 (63.5)	12/12 (100)	7/11 (63.6)
Medications, n (%)			
Diuretics	7/85 (8.3)	0/12 (0)	0/11 (0)
ASA	5/85 (5)	0/12 (0)	0/11 (0)
ACE inhibitors	17/85 (20)	0/12 (0)	0/11 (0)
Statins	14/85 (17.6)	2/12 (20)	3/8 (37.5)

ACE indicates angiotensin‐converting enzyme; ASA, acetylsalicylic acid; COPD, chronic obstructive pulmonary disease.

We tested whether Rac1 inhibition could restore the endothelial function of dysfunctional saphenous veins. NSC23766, a pharmacological inhibitor of Rac1,^28^ was able to significantly improve endothelium‐dependent vasorelaxation of varicose venous portions, whereas it was ineffective in nonvaricose ones (Figure 1C and 1D).

**Figure 2:**
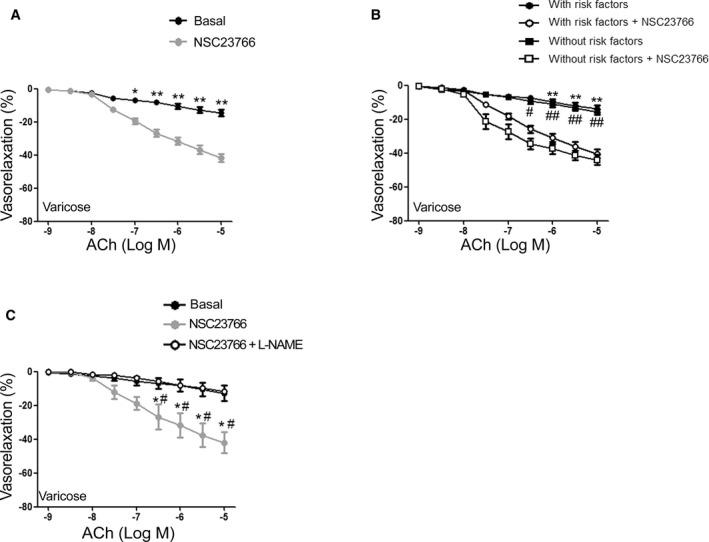
Rac1 inhibition improves endothelial function of human saphenous veins. A, Endothelial‐dependent vasorelaxation of varicose human saphenous veins before (Basal) and after treatment with Rac1 inhibitor (NSC23766). Data are given as mean±SEM (n=56). *P<0.05; **P<0.01. B, Dose‐response curves of varicose saphenous veins from patients with or without risk factors in response to increasing doses of acetylcholine (ACh) at baseline and after exposure to NSC23766. Data are given as mean±SEM (n=9 no risk factors; n=47 with risk factors). **P<0.01 vs with risk factors plus NSC23766; ^#^ *P*<0.05; ^##^ *P*<0.01 vs without risk factors plus NSC23766. C, Dose‐response curves of human saphenous veins in response to increasing doses of acetylcholine (ACh) before (Basal), after NSC23766 treatment, or after NSC23766 plus L‐NAME treatment. Data are given as mean±SEM (n=8). *P<0.05 vs Basal; ^#^ *P*<0.05 vs NSC23766+L‐NAME. L‐NAME refers to L^G^‐nitro‐l‐arginine.

Remarkably, we corroborated these data on the saphenous veins harvested from the entire study population of patients with chronic venous insufficiency and/or varicose veins and confirmed that NSC23766 could restore the endothelial function of altered saphenous vein segments. Interestingly, pharmacological Rac1 inhibition improved vasorelaxation of veins collected from patients without cardiovascular risk factors and from subjects with risk factors in a similar manner (Figure 2A and 2B). These beneficial effects were found to be nitric oxide dependent because L‐NAME, an inhibitor of NO production, abrogated the vasorelaxant effects of NSC23766 (Figure 2C). Overall, the data indicate that endothelial dysfunction observed in altered saphenous vein segments can be reversed by Rac1 inhibition and suggest that Rac1 plays a pivotal role in the genesis of venous endothelial dysfunction in humans. Of note, in a different setting of experiments conducted on mouse vessels (Data S1), we found that NSC23766 at the dose of 30 μmol/L does not influence acetylcholine‐evoked vasorelaxation (Figure S1). These data exclude the possibility that NSC23766 can directly interfere with muscarinic receptors, as previously reported for higher concentrations in cardiomyocytes.^29^

**Figure 3:**
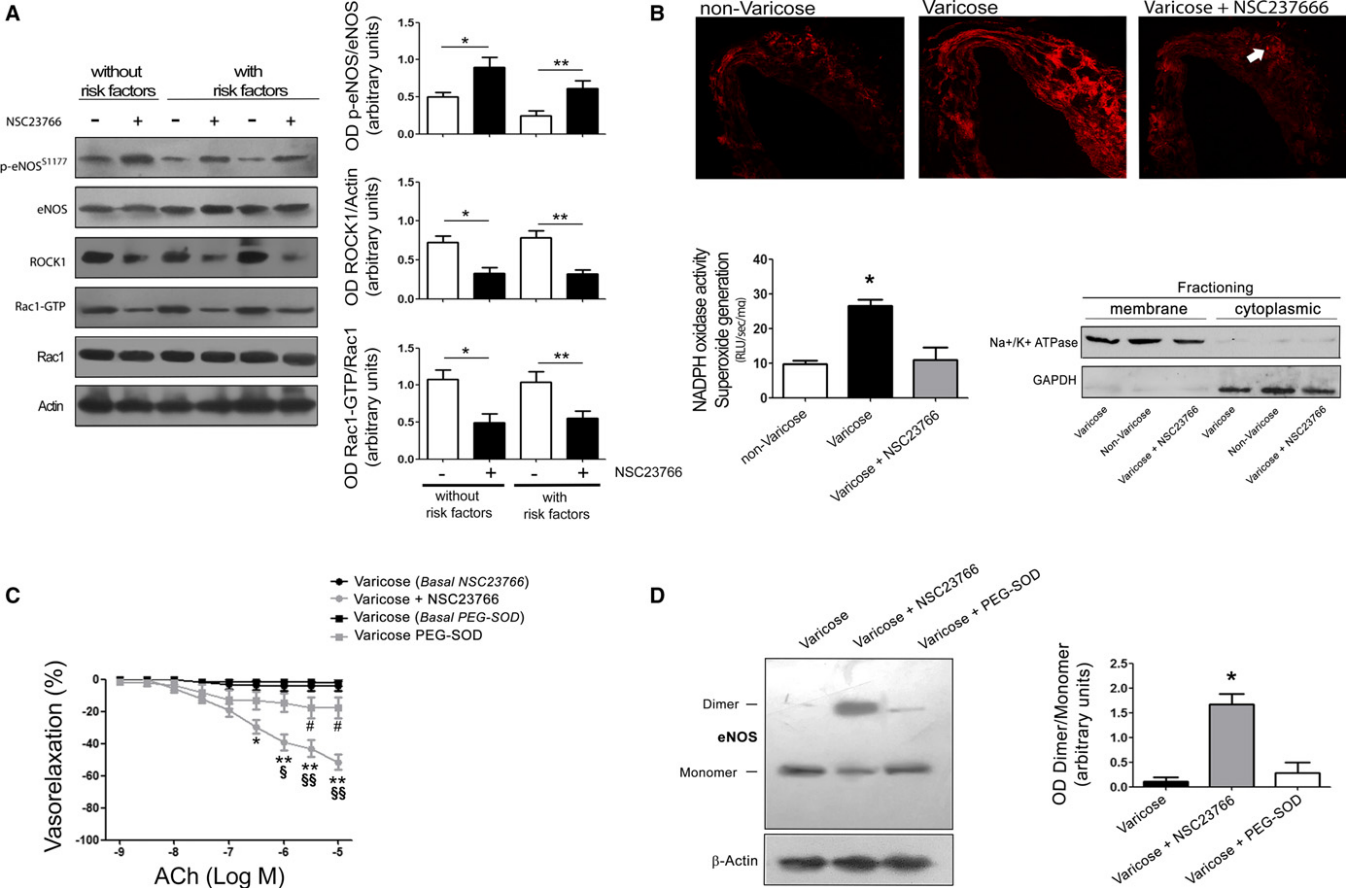
Rac1 inhibition increases endothelial nitric oxide synthase (eNOS) phosphorylation and reduces oxidative stress. A, Representative immunoblot analysis from varicose human saphenous veins collected from patients with or without risk factors. Data are given as mean±SEM; *P<0.05 (n=6). Symbols: −, without NSC23766; +, in presence of NSC23766. B, Top: In situ detection of superoxide generation in segments of human nonvaricose and varicose saphenous veins collected from the same patients. Bottom: Graphs of superoxide production in membrane fraction measured continuously in segments of human nonvaricose and varicose saphenous veins collected from the same patients by using 20 μmol/L lucigenin‐enhanced chemiluminescence. Values are mean±SEM, expressed as RLU/(s·mg dry weight) (n=4). Right: Immunoblot showing the purity of fractions. C, Dose‐response curves of human varicose saphenous veins in response to increasing doses of acetylcholine (ACh), before (Basal) and after treatment with Rac1 inhibitor (NSC23766) or with PEG‐SOD. Data are given as mean±SEM (n=4 NSC23766; n=9 PEG‐SOD). *P<0.05; **P<0.01 vs varicose; ^§^ *P*<0.05; ^§§^ *P*<0.01 vs varicose plus PEG‐SOD; ^#^ *P*<0.05 vs varicose. D, Representative immunoblot and bar graph depicting the ratio of dimer/monomer eNOS band intensity in human varicose saphenous veins that were or were not treated with NSC23766 or PEG‐SOD. (n=4). *P<0.005 vs all. PEG indicates polyethylene glycol; RLU, relative luminescence units; SOD, superoxide dismutase.

Then we conducted biochemical analyses on dysfunctional vein portions treated or not treated with NSC23766. First, we found that NSC23766 inhibits Rac1 activity in vein segments from patients both without and with cardiovascular risk factors, as indicated by the reduction of Rac1‐GTP content (Figure 3A). Rac1 inhibition increased the phosphorylated levels of eNOS (Figure 3A). It also downregulated ROCK1 in line with eNOS activation (Figure 3A). In fact, ROCK1 was previously found to reduce both eNOS phosphorylation and gene expression.^30^, ^31^ Of note, we observed that Rac1 inhibition significantly reduces superoxide production in dysfunctional veins, as indicated by DHE staining (Figure 3B). Rac1 inhibition also inhibited NOX‐dependent superoxide generation in membrane fractions (Figure 3B). Of note, in a different setting of experiments conducted on mouse vessels we found that NSC23766 does not affect the increase of DHE fluorescence in vessels exposed to hydrogen peroxide, which directly induces ROS formation independently of Rac1 activation. On the other hand, NSC23766 significantly reduced the increase of DHE fluorescence induced by angiotensin II, a well‐known activator of Rac1 and NOX.^32^ These results indicate that NSC23766 does not directly compete with the DHE assay, further corroborating the evidence that Rac1 inhibition is responsible for the reduction of superoxide anion levels (Figure S2).

Overall, these data indicate that Rac1 inhibition reduces NOX activity and ROS production, coherently with the established activating function of Rac1 toward this ROS‐generating enzyme. Interestingly, we found that PEG‐SOD modestly but significantly improved acetylcholine‐induced vasorelaxation (Figure 3C). These data suggest that a reduction of oxidative stress may play a significant role in the beneficial effects of Rac1 inhibition on endothelial function. However, the effects of PEG‐SOD appeared to be significantly weaker than those exerted by NSC23766 (Figure 3C). We also observed that Rac1 inhibitor reduces eNOS uncoupling, as evaluated by the eNOS ability to form a homodimer,^25^ whereas PEG‐SOD did not seem to exert similar effects (Figure 3D).

**Figure 4:**
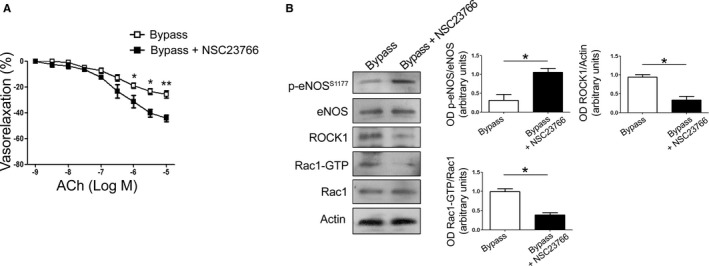
Rac1 inhibition improves endothelial function of human saphenous veins used to perform bypass. A, Dose‐response curves of human saphenous veins used to perform bypass in response to increasing doses of acetylcholine (ACh) before (Basal) and after treatment with Rac1 inhibitor (NSC23766). Data are given as mean±SEM (n=11; n=4 untreated; n=7 treated with NSC23766). *P<0.05; **P<0.01. B, Representative immunoblot analysis in protein samples from portions of human saphenous veins used to perform the bypass, before and after exposure to Rac1 inhibitor (NSC23766) in ex vivo experiments. Data are given as mean±SEM (n=4); *P<0.05. OD indicates optical desnsity; p‐eNOS, p‐endothelial nitric oxide synthase; ROCK1, RhoAassociated kinase 1.

Finally, we tested whether Rac1 inhibition is able to improve endothelial function of portions of saphenous veins used as conduits for surgical revascularization (bypass) of patients with peripheral ischemia due to severe artery disease (Table). In fact, the presence of vascular disease in these subjects implies a systemic endothelial dysfunction. NSC23766 significantly increased endothelium‐dependent vasorelaxation in response to acetylcholine and reduced NOX activity, thereby indicating that Rac1 inhibition improves endothelial function of saphenous vein grafts (Figure 4A). In addition, Rac1 inhibition restored eNOS phosphorylation levels and reduced ROCK1 expression consistently with what we observed in insufficient veins (Figure 4B).

## Discussion

In the present study we explored the effects of pharmacological Rac1 inhibition on human endothelial dysfunction. We found that Rac1 inhibition significantly improves endothelial function in dysfunctional saphenous veins collected from subjects with chronic vein insufficiency. Such beneficial effects were nitric oxide–dependent. Mechanistically, Rac1 inhibition reduced NOX activity and superoxide production. In addition, it downregulated ROCK1 and promoted eNOS upregulation. We also found that Rac1 inhibition improves endothelial function of saphenous vein grafts collected from patients who underwent bypass surgery for peripheral ischemia due to severe vascular disease.

Rac1 belongs to the Rho family of GTPases.^17^, ^18^ It is deeply involved in the regulation of multiple cellular processes such as cellular growth and proliferation. It also regulates cytoskeletal architecture, cellular migration, adhesion, and polarity.^17^, ^18^ Rac1 is highly expressed in endothelial cells, where it controls survival, proliferation, and angiogenesis. It also regulates endothelial cell response to shear stress.^17^, ^18^ Rac1 gene deletion in endothelial cells is associated with embryonic lethality due to multiple vascular defects.^33^, ^34^ This indicates that Rac1 exerts important endothelial physiological functions. However, Rac1 also contributes to maladaptive vascular processes. Rac1 is activated in response to vascular stress and thereby promotes vascular inflammation and oxidative stress.^17^, ^18^ It stimulates ROS production by inducing NOX activation in endothelial cells.^17^, ^18^ It also favors the recruitment of p67phox to the plasma membrane, where it binds to the other NOX subunits, thereby stimulating the NOX enzymatic function. Importantly, we previously found that Rac1 is activated in response to diabetes mellitus and mechanical stress in endothelial cells and is required for the development of endothelial dysfunction under these conditions.^19^, ^20^

We believe that our study significantly extends this evidence by demonstrating for the first time that that Rac1 also plays an important role in the development of human endothelial dysfunction. We found that Rac1 inhibition restores the endothelial function of dysfunctional saphenous vein segments in a nitric oxide–dependent manner. In fact, the vasorelaxant effects of NSC23766 were abrogated by L‐NAME. These data suggest that Rac1 pharmacological inhibition may increase nitric oxide bioavailability. Mechanistically, Rac1 inhibition may reduce nitric oxide degradation by reducing NOX activity and production of superoxide, which rapidly reacts with nitric oxide to form peroxynitrite.^1^, ^2^ Consistently, we found that Rac1 inhibitor reduced NOX‐dependent ROS production. Rac1 may regulate NOX1, NOX2, and NOX5 in endothelial cells. Future studies are warranted to understand if all these subunits contribute to Rac1‐induced endothelial dysfunction in a similar manner or whether there is a specific isoform that predominantly mediates the detrimental vascular effects of Rac1. Interestingly, we found that the beneficial vascular effects of NSC23766 in dysfunctional veins are much stronger than those exerted by PEG‐SOD. These data suggest that a selective inhibition of Rac1 would represent a more efficacious intervention to improve endothelial function with respect to a nonselective reduction of superoxide anion levels with scavengers.

Rac1 inhibition may directly increase nitric oxide production.^1^, ^2^ We found that NSC23766 leads to an increase of eNOS phosphorylation, the enzyme synthesizing nitric oxide. This mechanism appears to be likely mediated by the reduction of ROCK1, a negative regulator of eNOS. In fact, ROCK1 inhibits eNOS gene expression and inhibits AKT, which phosphorylates and activates eNOS.^30^, ^31^ The mechanisms through which Rac1 regulates ROCK1 expression levels remain to be clarified. The mechanisms promoting Rac1 activity in dysfunctional veins also warrant further elucidation. Rac1 is activated in response to mechanical stress through ILK1‐dependent mechanisms in endothelial cells.^20^ It would be interesting to investigate whether ILK1 contributes to Rac1 activation in insufficient saphenous veins as well. In addition, NSC23766 inhibits Rac1 activity by preventing its activation by the guanine nucleotide exchange factors TrioN and Tiam1.^28^ This evidence suggests that TrioN and Tiam1 may be directly involved in Rac1 activation in dysfunctional veins.

Our study suggests that Rac1 activation may play a role in vein‐related diseases. Recent evidence indicates that endothelial dysfunction contributes to vein‐related diseases. A procoagulative state, which is usually associated with endothelial dysfunction, may favor deep vein thrombosis.^1^, ^2^ In this regard Migliacci et al demonstrated that subjects with a history of spontaneous thromboembolism display endothelial dysfunction.^3^ Our study and previous work demonstrated that saphenous vein insufficiency is associated with a marked endothelial dysfunction.^5^ Finally, endothelial dysfunction accelerates the failure of saphenous vein grafts that are implanted in coronary and peripheral bypass surgery.^6^, ^7^, ^8^ Risk factors associated with early failure of venous bypasses are associated with a reduced endothelial function of saphenous vein grafts.^4^, ^9^, ^10^ In addition, elevated pressures of distension of saphenous veins during venous graft preparation are associated with endothelial damage and endothelial dysfunction, which can favor the early failure of the graft.^7^, ^35^ Rac1 may be involved in all these conditions. Rac1 activation promotes vascular inflammation, oxidative stress, and increased permeability, thereby favoring thrombosis.^17^, ^18^ In addition, Rac1 inhibition was shown to inhibit platelet aggregation. Rac1 is also activated by diabetes mellitus and high vessel distension pressure,^17^, ^18^, ^19^, ^20^ suggesting its potential involvement in venous graft failure. In support of this latter hypothesis, we found that Rac1 inhibition significantly improves the endothelial function of saphenous vein grafts used for peripheral bypass surgery. Therefore, we speculate that Rac1 might be a potential therapeutic target able to reduce the chances of closure of saphenous vein grafts used for surgical revascularization of both coronary and peripheral arteries.

Our study also has some limitations that should be acknowledged. First, Rac1 pharmacological inhibitor may have some nonspecific effects, although we confirmed that this compound significantly reduces Rac1 activation and NOX activity. We only had a small group of healthy vein portions. However, we were able to make a direct comparison of healthy versus diseased vein tracts from the same saphenous veins, which allowed us to demonstrate that varicose veins have endothelial dysfunction and significant Rac1 activation. Finally, DHE‐ and lucigenin‐based assays may not be completely specific for superoxide anion detection, although these are widely used and validated methods, and both of them provided similar results, which were also consistent with the Rac1‐GTP and eNOS uncoupling data.

In conclusion, our study demonstrates that Rac1 pharmacological inhibition rescues endothelial function and reduces oxidative stress in dysfunctional saphenous veins. Accordingly, these emerging data may have applicative/translational implications because Rac1 inhibition may represent a potential therapeutic intervention to reduce human endothelial dysfunction and subsequently vascular diseases in several clinical scenarios, particularly vein‐related diseases. Of course, future studies are encouraged to test this hypothesis and to confirm our results, testing the efficacy of Rac1 inhibition in other models of human endothelial dysfunction, particularly in human arteries.

## Sources of Funding

This study was financially supported by University of Rome Sapienza to Giacomo Frati, from Ministero della Salute to Sebastiano Sciarretta and Roberto Carnevale (GR‐2013‐02355401). The funding source had no involvement in the research design and preparation, the writing, or the decision to submit this article for publication.

## Disclosures

None.

## Supporting information


**Data S1.** Vascular Reactivity Studies
**Figure S1.** A, Acetylcholine (ACh) vasorelaxation in preconstricted mice mesenteric arteries in basal condition (Ctrl, full circles) and after treatment with Rac‐1 inhibitor at 30 μmol/L for 30 minutes (Ctrl+NSC23766, full square) (n=4 for each group). B, Nitroglycerin (Nitro) vasorelaxation in preconstricted mouse mesenteric arteries in basal condition (Ctrl, full circle) and after treatment with Rac‐1 inhibitor at 30 μmol/L for 30 minutes (Ctrl+NSC23766, full square) (n=4 for each group).
**Figure S2.** In situ detection of superoxide generation in segments of mouse mesenteric arteries treated with H_2_O_2_ (5 μmol/L); with H_2_O_2_ plus NSC23766 (30 μmol/L); with angiotensin II (ANG II; 1 μmol/L); or with angiotensin II plus NSC23766.
