# Supplementary material for: Rac1 Pharmacological Inhibition Rescues Human Endothelial Dysfunction
Source: J Am Heart Assoc. 2017 Feb 28;6(3):e004746. doi: 10.1161/JAHA.116.004746 (PMC5524008; doi:10.1161/JAHA.116.004746)

Supplemental Material

Data S1.

Vascular reactivity studies

All experiments involving animals were conformed to the Guide for the Care and Use of Laboratory Animals published by the US National Institutes of Health (NIH Publication No. 85-23, revised 2011) and were approved by IRCCS INM Neuromed review board.

Vessels were excised from mice and placed in a wire myograph system filled with Krebs solution. First, an analysis of vascular reactivity curves was performed. In particular, vasoconstriction was assessed with 80 mmol/L of KCl or with increasing doses of phenylephrine (from 10^{-9} M to 10^{-6} M) in basal conditions. Vascular responses were then tested before and after NSC23766 treatment (30µM). Endothelium-dependent and -independent relaxation was assessed by measuring the dilatory responses of mesenteric arteries to cumulative concentrations of acetylcholine (from 10^{-9} M to 10^{-5} M) or nitroglycerine (from 10^{-9} M to 10^{-5} M), respectively, in vessels pre-contracted with phenylephrine at a dose necessary to obtain a similar level of pre-contraction in each ring (80% of initial KCl-evoked contraction).

Supplemental Figures:

Figure S1. A) Acetylcholine (ACh) vasorelaxation in preconstricted mice mesenteric arteries in basal condition (Ctrl, full circle) and after treatment with Rac-1 inhibitor at 30 µM for 30 minutes (Ctrl+NSC23766, full square) (n=4 for each group). **B)** Nitroglycerine (Nitro) vasorelaxation in preconstricted mice mesenteric arteries in basal condition (Ctrl, full circle) and after treatment with Rac-1 inhibitor at 30 µM for 30 minutes (Ctrl+NSC23766, full square) (n=4 for each group).

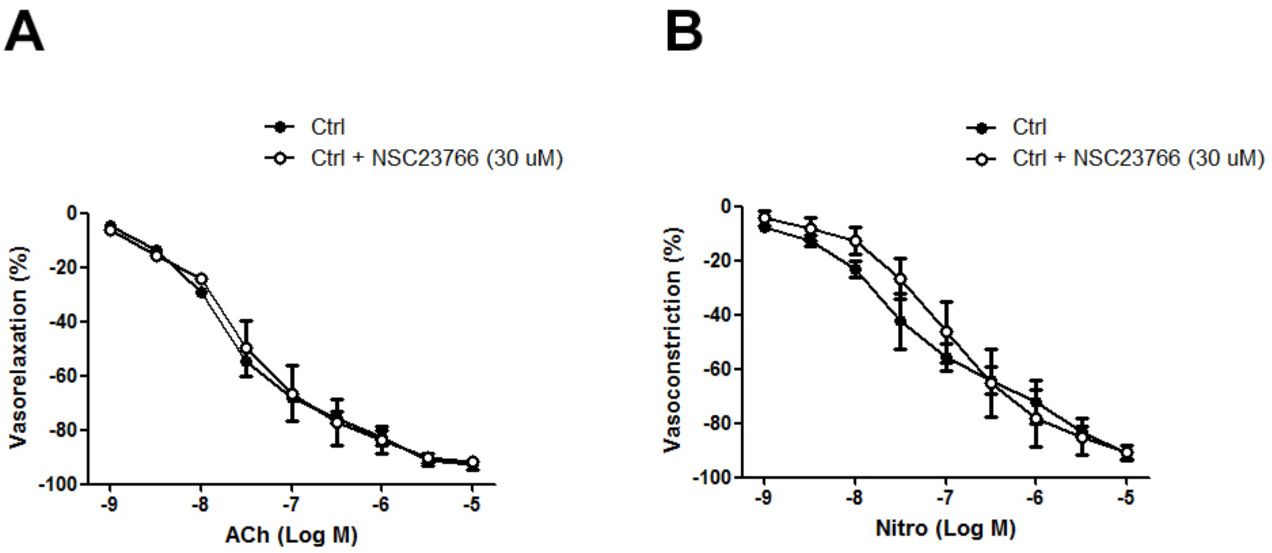

Figure S2. In situ detection of superoxide generation in segments of mouse mesenteric arteries treated with H_2O_2 (5 µM); with H_2O_2 plus NSC23766 (30 µM); with Angiotensin II (ANG II; 1 µM) or with Angiotensin II plus NSC23766.

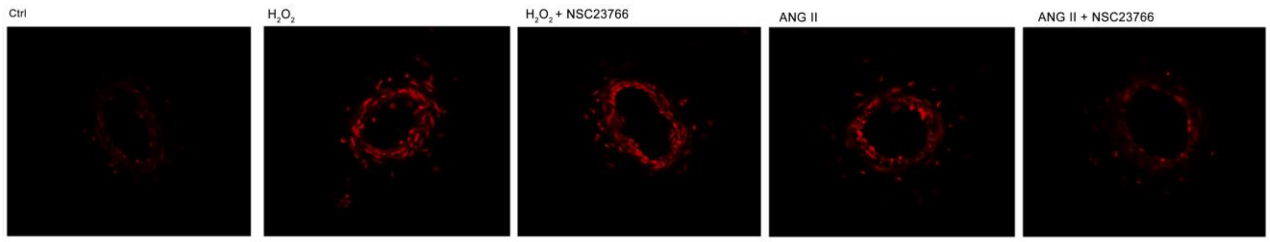

Supplement: Supplementary file 1 — Data S1. Vascular Reactivity Studies Figure S1. A, Acetylcholine (ACh) vasorelaxation in preconstricted mice mesenteric arteries in basal condition (Ctrl, full circles) and after treatment with Rac‐1 inhibitor at 30 μmol/L for 30 minutes (Ctrl+NSC23766, full square) (n=4 for each group). B, Nitroglycerin (Nitro) vasorelaxation in preconstricted mouse mesenteric arteries in basal condition (Ctrl, full circle) and after treatment with Rac‐1 inhibitor at 30 μmol/L for 30 minutes (Ctrl+NSC23766, full square) (n=4 for each group). Figure S2. In situ detection of superoxide generation in segments of mouse mesenteric arteries treated with H2O2 (5 μmol/L); with H2O2 plus NSC23766 (30 μmol/L); with angiotensin II (ANG II; 1 μmol/L); or with angiotensin II plus NSC23766. [file JAH3-6-e004746-s001.pdf]
